# Maternal Substance Use: Consequences, Identification, and Interventions

**DOI:** 10.35946/arcr.v40.2.06

**Published:** 2020-06-30

**Authors:** Grace Chang

**Affiliations:** 1 U.S. Department of Veterans Affairs Boston Healthcare System, Boston, Massachusetts; 2 Harvard Medical School, Department of Psychiatry, Boston, Massachusetts

**Keywords:** prenatal alcohol substance use, screening and intervention

## Abstract

Alcohol, tobacco, and cannabis are the substances most frequently used during pregnancy, and opioid-exposed pregnancies have increased fourfold. The purpose of this review is to describe the prevalence and consequences of prenatal exposure to alcohol, tobacco, cannabis, and opioids. Currently available screening questionnaires for prenatal substance use are summarized and contrasted with the measures available for prenatal alcohol use. Because screening for prenatal alcohol and substance use is but the prelude to efforts to mitigate the potential adverse consequences, attempts for the modification of these consequences are briefly reviewed. In addition, areas of future research related to the criminalization of prenatal substance use, which may inhibit both inquiry and disclosure, are discussed. Indeed, the full potential of effective interventions has yet to be realized.

## INTRODUCTION

Prenatal exposure to alcohol and other substances has become increasingly common. The substances used most frequently during pregnancy are alcohol, tobacco, and cannabis. Moreover, between 1999 and 2014, the number of women with opioid use disorder during labor and delivery quadrupled.^1^ The purpose of this review is to describe the prevalence and consequences of prenatal exposure to alcohol, tobacco, cannabis, and opioids. Currently available screening questionnaires for prenatal substance use are summarized and contrasted with the measures available for prenatal alcohol use. Because screening for prenatal alcohol and substance use is but the prelude to efforts to mitigate the potential adverse consequences, attempts for the modification of these consequences are also briefly reviewed.

It should be noted that this review article is not intended to be a systematic review of the world literature on either prenatal substance use or its prevention. Rather, it is a narrative literature review that is meant to be illustrative and to stimulate areas of future research because the full potential of effective interventions has yet to be realized.

## THE CONSEQUENCES OF PRENATAL SUBSTANCE USE

The consequences of prenatal substance use differ depending on the specific substances used. The most commonly used substances include alcohol, tobacco, cannabis, and opioids.

### Prenatal Alcohol Use and Its Consequences

The estimated percentage of prenatal alcohol use is approximately 15%, with past month use being approximately 13%.^2^,^3^ A Centers for Disease Control and Prevention survey conducted from 2015 to 2017 found that nearly 4% of pregnant women had engaged in binge drinking in the prior 30 days.^4^ Alcohol use during pregnancy is a highly preventable cause of birth defects and developmental disabilities.^5^ Despite the recognition of the teratogenic properties of alcohol, many women continue to disregard advisories on avoiding alcohol during pregnancy.^6^

There is no known safe level of alcohol use while pregnant because there is no exact dose-response relationship between the amount of alcohol consumed during the prenatal period and the extent of damage caused by alcohol in the fetus.^7^ Thus, an infant born to a mother who drank alcohol while pregnant may be normal or may manifest alcohol-related birth defects (e.g., problems with the heart, kidneys, bones, or hearing), alcohol-related neurodevelopmental disorders (e.g., intellectual disabilities or problems with behavior and learning), or fetal alcohol spectrum disorders (FASD), which includes a wide range of effects, from mild to severe. An individual with FASD might have abnormal facial features; small head size; shorter than average height; low body weight; poor coordination; hyperactive behavior; difficulty with attention; poor memory; difficulties in school, especially with mathematics; learning disabilities; speech and language delays; intellectual disability or low IQ; poor reasoning and judgment skills; sleep and sucking problems as a baby; vision or hearing problems; and problems with the heart, kidneys, or bones.^8^

A recent multisite study using active case ascertainment methods estimated that the prevalence of FASD among first graders ranged from 1% to 5%.^9^ This is concerning because these disorders are associated with life-long disabilities. However, early intervention treatment services can improve a child’s development and function.^8^

There is continuing uncertainty about the effects of low and low-to-moderate levels of alcohol intake during pregnancy.^10^ For example, a recent cohort study reported craniofacial changes with almost any level of prenatal alcohol intake, but the clinical significance of these changes is not known.^11^ Factors that may influence the effects of prenatal alcohol use include patterns of maternal drinking, maternal and fetal genetics, as well as socioeconomic and ethnic factors. Because there is no proven “safe” level of alcohol exposure during pregnancy, the most prudent advice for pregnant women is to abstain from drinking.^12^

### Prenatal Tobacco Use and Its Consequences

Cigarette smoking in the antepartum period is common. Past month use of tobacco products among pregnant women was approximately 15% according to the 2017 National Survey on Drug Use and Health report.^13^ Tobacco products include the use of alternative forms of nicotine, such as e-cigarettes and vaping, which until recently, have been perceived to be less harmful. For example, in 2015, as many as 7% of women with a recent live birth in Oklahoma and Texas reported using an electronic vapor product shortly before, during, or after pregnancy.^14^ Data specific to the effects of prenatal use of electronic vapor products are sparse. However, the Centers for Disease Control and Prevention has issued interim guidance that electronic cigarette products should never be used by pregnant women or adults who do not currently use tobacco products as it investigates the more than 200 cases of severe pulmonary disease associated with their use.^15^

The use of any tobacco product during pregnancy is associated with adverse maternal, fetal, and neonatal outcomes. Examples of the adverse consequences of tobacco use may begin with subfertility and delay in conception among women who smoke and extend to pregnancy outcomes, which include increased risk of spontaneous pregnancy loss, placental abruption, preterm premature rupture of membranes, placenta previa, preterm labor and delivery, low birth weight, and ectopic pregnancy. Prenatal cigarette smoking may exert effects beyond pregnancy as well and is associated with increased risks of asthma, infantile colic, and childhood obesity.^16^

### Prenatal Cannabis Use and Its Consequences

Past month cannabis use among pregnant women ages 18 to 44 increased between 2002 and 2017 from approximately 3% to 7%.^17^ Among pregnant adolescents, past month use (15%) was even higher.^18^ A recent cross-sectional study using data from 367,403 pregnancies among 276,991 women in Northern California found that self-reported daily, weekly, and monthly cannabis use before and during pregnancy increased between 2009 and 2017. The greatest increases were for daily use, reaching 25% among those who used in the year before pregnancy and 21% among those who used during pregnancy.^19^ Explanations for the increases in prenatal use include increasing acceptance of cannabis use and decreasing perceptions of cannabis-related harms.^20^

The association between prenatal cannabis use and maternal, perinatal, and neonatal outcomes is unclear.^21^ A 2016 systematic review and meta-analysis concluded that maternal marijuana use during pregnancy was not an independent risk factor for adverse neonatal outcomes, such as low birth weight or preterm delivery, after adjusting for confounding factors like tobacco use.^22^ However, limitations to the generalizability of this meta-analysis include the relatively few women in the risk-adjusted group, indicating that the meta-analysis was underpowered to stratify for all secondary outcomes of interest. Another systematic review and meta-analysis from the same time frame found that pregnant women who used marijuana had increased odds of being anemic and that infants exposed to cannabis in utero had decreased birth weight and were more likely to require neonatal intensive care.^23^ The researchers from this review acknowledged that because many cannabis users often use tobacco and alcohol as well, discerning a cannabis-only effect was not possible. A population-based cohort study of 661,617 women in Ontario, Canada, showed that the percentage of preterm births among self-reported cannabis users was 12% compared to 6% among nonusers, with this increase persisting even after adjusting for confounding factors.^24^ Until there is definitive evidence demonstrating the safety of prenatal marijuana use, concerns that marijuana may interfere with neurodevelopment as well as have other effects have resulted in the American College of Obstetricians and Gynecologists (ACOG) advising women who are pregnant or thinking about pregnancy to avoid using marijuana and other cannabinoids.^25^

### Prenatal Opioid Use and Its Consequences

Opioid use among pregnant women increased fourfold between 1999 and 2014 and is present in approximately 3% of pregnancies.^26^ Women who use opioids during pregnancy are a diverse group because opioid use may occur in the context of medical care, opioid misuse, or untreated opioid use disorder.^27^

Prenatal opioid use can have a far-reaching clinical impact on infant outcomes. Infants with prenatal opioid exposure are typically born smaller and may have neonatal opioid withdrawal syndrome (NOWS). Infants with NOWS experience withdrawal from opioids and require additional medical care.^28^ Characteristics of NOWS, also known as neonatal abstinence syndrome (NAS), include disturbances in gastrointestinal, autonomic, and central nervous systems, leading to irritability, high-pitched crying, poor sleep, and uncoordinated sucking reflexes that lead to poor feeding. In 2014, a baby was born with NOWS in the United States every 15 minutes.^29^,^30^

The full impact of opioid exposure during pregnancy on fetal, infant, and childhood outcomes, however, is still unknown. Explanations include the possibility of exposure to other substances as well as concomitant maternal, medical, psychological, and socioeconomic issues. There is a growing body of evidence about the association of opioids with specific birth defects, such as congenital heart defects, neural tube defects, and clubfoot.^31^

For pregnant women with opioid use disorder, substitution treatment with opioid agonists, such as methadone and buprenorphine, imparts important benefits particularly when compared to continued illicit drug use. Advantages include more stable maternal drug levels, reduced withdrawal and drug-seeking behavior, and improved self-care, which should lead to a better pregnancy outcome because of reduced risk for fetal distress, miscarriage, growth restriction, and preterm birth.^32^

Compared to data on buprenorphine-maintained pregnancies, more longitudinal data on methadone-exposed pregnancies are available. In a prospective longitudinal study, 68 methadone-exposed children and 88 nonmethadone-exposed children were evaluated at 2.0 and 4.5 years for executive functioning and later emotional behavioral and emotional adjustment.^33^ The methadone-exposed children had worse inhibitory control than the nonexposed children, when taking maternal education and prenatal benzodiazepine use into account. Another study used a school readiness framework to assess the health and neurodevelopmental outcomes of a regional cohort of 100 methadone-exposed children and 110 randomly identified nonmethadone-exposed children who were studied from birth to 4.5 years. Children born to opioid-dependent mothers had higher rates of delay and impairment across all outcome domains, with multiple domain problems being common. Impaired school readiness was associated with greater maternal substance use, higher social risk, male sex, and lower quality caregiving environments.^34^

A systematic review and meta-analysis synthesized data from 41 studies on the neurodevelopment of prenatal methadone-exposed children. The analysis included 1,441 children whose mothers were prescribed methadone during pregnancy and 842 children whose mothers did not receive methadone.^25^ Methadone-exposed children appeared to be at increased risk for neurodevelopmental impairment, with lower scores on the Mental Development Index and Psychomotor Development Index, as well as atypical visual evoked potentials, strabismus, and nystagmus. However, these findings about impairment may be biased, with the studies not accounting for factors other than methadone. Indeed, results from this meta-analysis confirm the need for more research and the many factors that can impact pregnancy outcome.

## SCREENING FOR PRENATAL SUBSTANCE USE

Early universal screening of pregnant women for alcohol use, substance use, or both is recommended by ACOG because alcohol and substance use is not typically disclosed spontaneously by patients. ACOG recommends clinicians use validated questionnaires or have a conversation with patients but does not endorse using routine urine toxicology tests.^35^,^36^ Moreover, a positive screening questionnaire does not result in a diagnosis. Rather, such a result is an opportunity for a patient and her clinician to review health practices and make changes, if appropriate.^37^

### Screening for Prenatal Alcohol Use

There is no known safe level of alcohol consumption during pregnancy.^38^ Alcohol is a teratogen; in other words, it is capable of interfering with fetal development, resulting in birth defects. Although the consequences of light alcohol use among women, defined as consuming up to 32 g of alcohol per week, on pregnancy outcomes remain unsettled in the absence of sufficient evidence, the potential for harm cannot be ruled out.^12^ Hence, ACOG has recommended that all women seeking obstetric–gynecologic care be screened for alcohol use annually and within the first trimester of pregnancy.

Screening questionnaires for prenatal alcohol use have been well studied. For example, a systematic review of brief screening questionnaires to identify problem drinking during pregnancy evaluated seven instruments given to 6,724 participants.^39^ The measures included the TWEAK (Tolerance, Worried, Eye-Opener, Amnesia, K/Cut Down); the T-ACE (Tolerance [number of drinks], Annoyance, Cut Down, Eye-Opener); CAGE (Cut Down, Annoyed, Guilty, Eye-Opener), NET (Normal Drinker, Eye-Opener, Tolerance); AUDIT (Alcohol Use Disorder Identification Test); AUDIT-C (AUDIT Alcohol Consumption Questions), and SMAST (Short Michigan Alcoholism Screening Test). The screening questionnaires were compared with a structured interview to ascertain drinking status as a reference standard. The T-ACE, AUDIT-C, and TWEAK were the three questionnaires identified to be the most promising screening tools for identifying risk drinking in pregnant women. However, the sensitivity and specificity of these three questionnaires outside the United States is unknown.

### Screening for Prenatal Substance Use

Screening instruments for prenatal alcohol use have been well studied, whereas screening instruments for substances other than alcohol have been less well developed.^26^,^40^ The World Health Organization (WHO) guidelines for the identification and management of substance use and substance use disorder during pregnancy list the Substance Use Risk Profile-Pregnancy (SURP-P) scale,^41^ the proprietary 4P’s Plus ^©^,^42^ and the National Institute on Drug Abuse (NIDA) Quick Screen–Modified Alcohol, Smoking, and Substance Involvement Screening Test (ASSIST)^43^ as potential screening measures for pregnant women, even though not all of these instruments had been evaluated among that population at the time of its recommendation.^44^

Several recent studies have evaluated the accuracy of various screening tools for prenatal substance use. In one prospective cross-sectional study conducted in Baltimore, MD, with 500 pregnant women, stratified by trimester and use of prenatal care, researchers administered three index tests and compared them to reference tests.^45^ The three index tests were the proprietary 4P’s Plus^©^, NIDA Quick Screen–ASSIST), and the SURP-P. The reference tests were urine and hair testing, which captured substance use up to the past 90 days. Alcohol use was not evaluated. The researchers found that there were differences in validity indices (i.e., sensitivity, specificity, positive predictive value, and negative predictive value) by age and race, but not by trimester, for all screening tools. The SURP-P and 4P’s Plus^©^ were highly sensitive across all trimesters, races, and age groups.

Another prospective cross-sectional screening accuracy study compared five screening instruments on their ability to identify illicit drug, opioid, and alcohol use under privacy expectations consistent with current practice. The participants included 1,220 pregnant women who were receiving care in Boston, MA; Detroit, MI; or New Haven, CT. The women were socioeconomically diverse and had a mean age of 29 years. The study used a reference standard of substance use in three classes (i.e., illicit drugs, opioids, and alcohol); results were considered positive if use was evident via a 30-day calendar recall or urine toxicology analysis.^46^ The illicit drug use reference standard included marijuana, cocaine, heroin, amphetamines, barbiturates, and hallucinogens. The five screening instruments for substance use in pregnancy were the SURP-P; CRAFFT, a five-item screener with items related to car, relax, alone, forget, friends, and trouble; 5Ps, with items on parents, peers, partner, pregnancy, past (i.e., an adaptation of the 4P’s Plus^©^); Wayne Indirect Drug Use Screener (WIDUS); and NIDA Quick Screen–ASSIST. None of the five measures showed both high sensitivity and high specificity, and the area under the curve was low for nearly all measures, indicating that none could be recommended for applied practice with pregnant women.

A companion study compared the same five measures in the identification of substance use disorder, including alcohol, cannabis, opioids, and stimulants, among the 1,220 pregnant women.^47^ Participants completed the Mini International Neuropsychiatric Interview 7.0.2, a short, structured diagnostic interview to identify substance use disorder, including alcohol; cannabis; stimulants, such as cocaine or amphetamines; and opioids, such as heroin and the nonmedical use of prescription drugs.^48^ Substance use disorder is distinct from substance use and represents a more significant and persistent pattern of consumption that may increase the risk of adverse infant outcomes as well as indicate that the pregnant woman may need evaluation and referral for specialty treatment.^49^ Of the 1,220 women in this study, more than 15% satisfied diagnostic criteria for substance use disorder and more than 30% reported having used alcohol or other substances in the past month. There was little overlap between the women who had substance use disorder and the women who had used alcohol or other substances within the past month. Nearly 10% of the women satisfied criteria for alcohol use disorder, as defined in the fifth edition of the *Diagnostic and Statistical Manual of Mental Disorders*, and 9.0% satisfied criteria for substance use disorder. Specifically, cannabis use disorder was the most common substance disorder diagnosed (8%). Approximately 3% satisfied criteria for more than one disorder.

There were considerable variations by site. For example, alcohol use disorder was the most common in Boston (15%) but infrequent in New Haven (5%). In contrast, substance use disorder was the most common in Detroit (17%) but less frequent in Boston (3%). Measures of merit (i.e., sensitivity, specificity, accuracy, and area under the receiver operating curve [AUROC]) were calculated with 95% confidence intervals [CI] for the NIDA Quick Screen, CRAFFT, SURP-P, WIDUS, and 5Ps, using substance use disorder as the criterion standard. The CRAFFT (AUROC=0.75, 95% CI [0.72, 0.79]) and SURP-P (AUROC=0.74, 95% CI [0.71, 0.78]) had the highest AUROCs for identifying substance use disorder, including alcohol. In contrast, the NIDA Quick Screen had the lowest AUROC (AUROC=0.62, 95% CI [0.59, 0.65]) for identifying substance use disorder, including alcohol. Overall, the tested measures were more accurate in identifying alcohol use disorder than substance use disorder (e.g., for identifying alcohol use disorder, the AUROCs for the CRAFFT and SURP-P were 0.78 and 0.77, respectively).

### Barriers to Early Identification by Screening

Pregnant women with substance use disorder are at increased risk for adverse health and social outcomes, making early identification crucial.^50^ Because substance use is substantially underreported, even among women who participate regularly in urine drug screens, use of validated questionnaires to identify prenatal alcohol and substance use has been recommended.^26^,^51^

There are, however, at least two barriers to these recommendations. First, as discussed in the preceding section, current screening questionnaires have been found to be inadequate measures. According to a 2010 survey of obstetrician-gynecologists, 58% did not use a validated screening tool to assess alcohol risk despite there being several validated tools available.^52^ It is likely that even fewer will use a screening tool for prenatal substance use, particularly as such tools are less well developed. A second barrier includes the punitive consequences stemming from state laws regarding prenatal substance use, which can result in patients not wanting to disclose and physicians not wanting to learn about their patients’ behaviors.^53^–^55^ Hence, in addition to patients’ previous fears about stigmatization because of use, disclosure could now pose a legal risk.^56^ An example of a punitive policy includes treating substance use during pregnancy as child abuse or neglect. This policy may arise from a desire to discourage women from using substances while pregnant, to encourage women to seek treatment, and to ensure the safety of the neonate.^57^

The association between states with punitive or reporting policies related to substance use in pregnancy and rates of NAS was recently evaluated in a study of 4,567,963 births from 8 U.S. states in varying years between 2003 and 2014.^57^ States without punitive or reporting policies were compared with states that had such policies, before and after policy enactment. The main outcome measure was the rate of NAS. States that criminalized substance use during pregnancy (e.g., grounds for civil commitment, child abuse, or neglect) had significantly higher rates of NAS in the 1st full year after enactment and more than 1 full year after enactment. In contrast, there was no association with neonatal abstinence rates in states with policies requiring reporting of suspected prenatal substance use. A possible explanation for this difference includes the extent to which pregnant women disengage from health care services when punitive measures are enforced, whereas reporting policies may not dissuade pregnant women from engaging with health care services, resulting in greater conversations between physicians and their patients. However, neither the punitive nor the reporting approach resulted in reduced rates of NAS, which was the presumed, desired outcome of these policies.

## AFTER SCREENING: INTERVENTION

Because screening for prenatal alcohol and substance use is but the prelude to efforts to mitigate the potential adverse consequences, brief intervention and referral to treatment, if indicated, have also been recommended.^56^ Brief interventions and psychosocial interventions have been examined by investigators and organizations such as the WHO, which sought to develop evidence-based global guidelines for identifying and managing substance use and substance use disorder in pregnancy.^42^ Global guidelines were desired because although several high-income countries had developed national guidelines, low- and middle-income countries had not. However, the WHO noted that much of the evidence underlying the effectiveness of screening and brief interventions during pregnancy originated from a time when reporting standards and measures of bias were not in consistent use. Nonetheless, the evidence indicated that asking women about alcohol and other substance use in a detailed and comprehensive way may increase their awareness of the risks associated with these practices and prompt them to modify their behavior.

### Psychosocial Interventions for Prenatal Alcohol Use

In late 2018, the U.S. Preventive Services Task Force (USPSTF) renewed its recommendation for screening adults ages 18 year or older, including pregnant women, for unhealthy alcohol use and providing persons engaged in risky or hazardous drinking with brief behavioral counseling interventions to reduce unhealthy alcohol use (i.e., a grade B recommendation meaning that there is high certainty that the net benefit is moderate, or moderate certainty that the net benefit is moderate to substantial).^56^ The USPSTF bounds the harms of screening and brief behavioral counseling interventions for unhealthy alcohol use in adults as small to none, based on the likely minimal risks of completing screening questionnaires, the noninvasive nature of the interventions, and the absence of reported harms in the evidence of the behavioral interventions.

The USPSTF makes three special comments with regards to pregnant women. First, any alcohol use by pregnant women is unhealthy. Second, validated alcohol screening tools for pregnant women are available, including the T-ACE and TWEAK. Third, brief counseling interventions among pregnant women have increased the likelihood that women remain abstinent from alcohol use during pregnancy.

Most interventions for FASD have been reported in North America, which has lower FASD prevalence compared to Europe and other sites around the world.^57^ Context-related differences may impact on the effectiveness of the interventions. For example, in a systematic review of prevention interventions to reduce prenatal alcohol exposure and FASD in indigenous communities, reviewers evaluated studies conducted from 1989 to 2017. A total of 10 studies from an initial sample of 712 articles were included if inclusion criteria were met. Comparisons of study effects were made difficult by heterogenous study designs, target populations, and interventions. The reviewers concluded that there was minimal evidence to support the belief that interventions intended to reduce the risk of prenatal alcohol exposure or FASD in indigenous populations have been effective.^58^

### Psychosocial Interventions for Prenatal Cigarette Smoking

Psychosocial interventions for supporting women to stop smoking during pregnancy were assessed by the Cochrane Pregnancy and Childbirth Group.^59^ This review included 102 randomized controlled trials, with 120 intervention arms. Data from 88 randomized controlled trials, involving more than 28,000 women, were analyzed. Intervention strategies included counseling, health education, feedback, incentives, social support, and exercise. Nearly all studies were conducted in high-income countries. Results from the review yielded moderate- to high-quality evidence that psychosocial interventions increased the proportion of pregnant women who had stopped smoking by late pregnancy (35%), with a 17% reduction in infants born with low birth weight, and a 22% reduction in neonatal intensive care admissions. There did not appear to be any adverse psychological effects from the interventions.

### Psychosocial Interventions to Reduce Other Prenatal Substance Use

Screening, brief intervention, and referral to treatment in the perinatal period have been recommended for prenatal substance use.^60^ Subsequent to this recommendation, at least two systematic reviews of the evidence for psychosocial interventions have been completed.

The first systematic review included four articles published between 2002 and 2013. It began with 3,792 unique potential publications, but the vast majority did not meet a priori quality criteria. Limited, but promising, evidence of brief interventions reducing illicit drug use among postpartum women was found.^61^

The second systematic review was completed by researchers from the Cochrane Collaboration. They sought to evaluate the evidence on the effect of psychosocial interventions, such as contingency management (CM) and motivational interviewing-based (MIB) techniques compared to that of usual care for pregnant women in outpatient illicit drug treatment programs.^62^ This group reviewed 14 studies, with 1,298 pregnant women who received either CM or MIB techniques in addition to other comprehensive care. The women in the control group received usual care that included pharmacological management, counseling, prenatal care, transportation, and/or childcare. There were no differences in retention or abstinence behavior between CM/MIB techniques and usual comprehensive care. The quality of evidence from these studies was assessed to be low to moderate.

## SUMMARY

Prenatal exposure to alcohol, tobacco, and marijuana has become increasingly common. In addition, there has been a fourfold increase in the number of opioid-exposed pregnancies. Prenatal exposure to alcohol and other substances may have an adverse impact on a developing fetus. Since pregnant women may be reluctant to disclose their use or may not appreciate the potential for harm, early identification is desirable. However, identification is currently limited by the lack of adequate screening tools and the fear of legal and other sanctions, which may limit both inquiry and disclosure. Although effective interventions for prenatal alcohol, cigarette, and other substances are available, these interventions rely on identification and behavioral counseling. It is likely that the full potential of effective interventions cannot yet be realized in the current setting.
